# Demyelinating processes in aging and stroke in the central nervous system and the prospect of treatment strategy

**DOI:** 10.1111/cns.13497

**Published:** 2020-11-19

**Authors:** Di Chen, Yichen Huang, Ziyu Shi, Jiaying Li, Yue Zhang, Ke Wang, Amanda D. Smith, Ye Gong, Yanqin Gao

**Affiliations:** ^1^ Department of Critical Care Medicine and Neurosurgery of Huashan Hospital State Key Laboratory of Medical Neurobiology MOE Frontiers Center for Brain Science, and Institutes of Brain Science Fudan University Shanghai China; ^2^ Geriatric Research Education and Clinical Center Veterans Affairs Pittsburgh Health Care System Pittsburgh PA USA

**Keywords:** aging, demyelination, remyelination, stroke, white matter injury

## Abstract

Demyelination occurs in response to brain injury and is observed in many neurodegenerative diseases. Myelin is synthesized from oligodendrocytes in the central nervous system, and oligodendrocyte death‐induced demyelination is one of the mechanisms involved in white matter damage after stroke and neurodegeneration. Oligodendrocyte precursor cells (OPCs) exist in the brain of normal adults, and their differentiation into mature oligodendrocytes play a central role in remyelination. Although the differentiation and maturity of OPCs drive endogenous efforts for remyelination, the failure of axons to remyelinate is still the biggest obstacle to brain repair after injury or diseases. In recent years, studies have made attempts to promote remyelination after brain injury and disease, but its cellular or molecular mechanism is not yet fully understood. In this review, we discuss recent studies examining the demyelination process and potential therapeutic strategies for remyelination in aging and stroke. Based on our current understanding of the cellular and molecular mechanisms underlying remyelination, we hypothesize that myelin and oligodendrocytes are viable therapeutic targets to mitigate brain injury and to treat demyelinating‐related neurodegeneration diseases.

## Graphical abstract

Reviews of cellular or molecular mechanism of demyelination process and therapeutic strategies for remyelination via OPCs and other potential targets in aging and stroke.

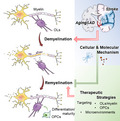

## 1 INTRODUCTION

In the central nervous system (CNS), myelin is formed and maintained by oligodendrocytes (OLs).^1^ The myelin sheath insulates the axon and facilitates salt conduction, which allows for rapid conduction of action potentials.^2^ Demyelinating diseases are a group of heterogeneous neurological diseases in which selective destruction to myelin primarily occurs. Many neurological diseases principally involve the process of demyelination, of which multiple sclerosis (MS) is the most common and typical. In diseases characterized by primary demyelination, the myelin sheaths and myelin supporting cells (such as OLs or Schwann cells) are damaged to varying degrees, while other components of CNS tissue remain relatively intact.^3^ However, it is important to note that although the damage to myelin is more extensive, demyelinating diseases always involve damage to other elements of the CNS. For example, damage to axons, which is highly correlated with neurological deficits,^4^ often occurs in demyelinating diseases. In addition, in focusing on overt myelin loss, microstructural changes in myelin are often ignored, but may indicate early‐stage demyelination.^5^ Therefore, loss of myelin sheaths and OLs alone does not fully represent comprehensive myelin‐related damage, and hence, the diagnosis of demyelination should be extended.

It is also important to note that demyelination is not limited to demyelinating diseases. Indeed, some neurodegenerative diseases such as Alzheimer's disease (AD) also involve demyelination.^6^ In addition, common brain injuries such as ischemic stroke are also accompanied by the destruction of myelin structure and apoptosis of OLs.^7^ OLs are extremely susceptible to ischemic stroke, and myelin sheath loss is the pathological hallmark of white matter stroke (WMS).^8^ Evidence also suggests that degeneration of OLs and oligodendrocyte precursor cells (OPCs) increases with aging, and researchers have observed demyelination and significant structural changes in myelin of aged rodents, monkeys, and human brain.^5^, ^9^, ^10^ Therefore, studying demyelinating pathogenesis in different CNS disorders may deepen our understanding and create possible therapies for these diseases.

## 2 CONSTRUCTION AND FUNCTION OF MYELIN AND DEMYELINATING PROCESSES

### 2.1 The composition and function of myelin

Myelin is critical in the nervous system of more highly evolved animals, such as vertebrates, as myelin ensures rapid and efficient nerve conduction. In addition, myelin plays an important role in the plasticity of neural networks.^11^ In the PNS, the myelin is composed of Schwann cells and, unlike oligodendrocytes, a Schwann cell forms only one segment of the axonal myelin. In the CNS, myelin is formed and maintained by OLs.^1^ Each OL forms myelin sheaths around multiple axons; thus, multiple myelin sheaths are sequentially formed by different OLs on the same axon.^11^ Myelin contains up to about 70% of various lipids. Of these, cholesterol provides stability to myelin by regulating membrane fluidity and permeability. Galactoceramide/sulfide is essential for the maintenance and stability of myelin. Gangliosides are involved in the interaction between axons and myelin. Inositol phosphate is involved in regulating cellular processes. Collectively, these lipids primary function is to maintaining the stability and longevity of the myelin, thus limiting the impact of the lack of individual lipids.^5^

The protein structure of the myelin sheath includes major dense line, intraperiod line, radial components, cytoplasmic regions, gap junctions, and axon‐glial junction and axon‐glial internodal domain.^12^ An electron microscopic study of the structure of myelin sheaths revealed that myelin has a characteristic periodic structure of alternating electron‐dense and lighter layers, known as major dense lines and intraperiod lines.^12^ The major dense lines consist of a tightly coalesced cytoplasmic surface, whereas the intraperiod lines comprise a tightly juxtaposed outer membrane.^11^ Myelin basic protein (MBP) occupies the surface between two adjacent cytoplasmic membranes to form the major dense line.^12^ The most important function of the myelin is action potential propagation. Myelin insulates axons and facilitates salt conduction, thus allowing action potentials to be rapidly conducted.^2^ Tight myelin increases the local resistance of the axon and decreases the membrane capacitance.^11^ Usually, we have assumed that neuronal synaptic interconnections are involved in the construction of neural circuits, but myelin also provides a pathway for neural circuits to control input timing, for example, alterations in individual anatomical parameters of myelinated axons can result in slowing or speeding action potential propagation of some axons relative to other axons, which is important for motor and sensory processing.^11^ Recent research suggests that myelin is involved in the control of many activities in development and adulthood and contributes to the acquisition of new motor skills.^2^ In addition, myelin also provides metabolic support to axons. The surface of the axon is disconnected from the nutrient‐rich extracellular environment, and therefore, the axon must bind to the myelin sheaths to obtain important metabolites.^11^

### 2.2 Demyelinating symptoms and diseases

The demyelination phenomenon occurs in both demyelinating diseases and other disorders, and demyelinating diseases primarily involve demyelination that does not damage axons. Demyelinating diseases can be categorized into inflammatory demyelinating disease caused by factors such as the autoimmune system or an infectious diseases, demyelinating, or nonmyelinating diseases with genetic background. Severe demyelinating processes also occur in aging, cerebral ischemia, and AD, and demyelination is highly relevant to the mechanisms and symptoms of these diseases, as well as the treatment and prognosis of the disease.^13^

Within inflammatory demyelinating diseases of autoimmune causes, MS is most prevalent and representative. MS is a chronic disease of the CNS in which immune‐mediated inflammation, demyelination, and subsequent axonal damage cause loss of motor and sensory function.^14^ Clinically, most patients with MS experience recurrent episodes of motor and cognitive deficits.^15^ The histopathology of MS is characterized by the formation of inflammatory demyelinating lesions with varying degrees of axonal damage and glial proliferation of astrocytes. Demyelinating plaques are present in white and gray matter, such as the cerebral or cerebellar cortex, and brainstem nuclei. The activity of the lesions is reflected by the continuous destruction of myelin, and secondary axonal and neuronal destruction is the main cause of permanent neurological deficits in MS patients.^1^

Inflammatory demyelinating diseases caused by infections diseases are relatively rare. In one such disease, subacute sclerosing panencephalitis (SSPE), white matter (WM) demyelination is one of the early symptoms, mainly affecting the temporal and parietal lobes. SSPE is a slowly progressive brain disorder caused by mutant measles virus. It is hypothesized that in SSPE, cross‐reactivity between viral and myelin antigens may contribute to the development of immune‐mediated inflammatory demyelination. Over time, inflammation decreases, but destructive changes become more widespread and are accompanied by extensive demyelination and widespread reactive astrocytosis. Patients' motor and language skills are reduced, and in the terminal stages, patients are in a vegetative state.^16^

Demyelinating disorders with genetic causes, such as Charcot‐Marie‐Tooth (CMT) disorder type 1B, the Déjérine‐Sottas syndrome, and congenital hypomyelination, the disorders are associated with muscle weakness and atrophy, sensory deficits, and skeletal deformities.^17^ Metabolic damage to myelin‐forming cells can lead to primary demyelination due to genetic defects in Schwann's cells and OLs.^13^

As previously noted, demyelination is not limited to demyelinating diseases. OLs and Schwann's cells are particularly susceptible to ischemic injury,^18^ so demyelination often occurs in ischemic strokes.^19^ Primary demyelination is a degeneration and loss of nerve myelin with relatively preserved axons due to congenital or acquired etiologies. Secondary demyelinating disorders represent a spectrum of white matter disease characterized by damage to neurons or axons with the resultant breakdown of myelin. They are both frequently observed in traumatic brain injuries, AD, and aging. The symptoms of these disorders are also often accompanied by reduced cognitive and motor functions. And we will describe the mechanisms and therapeutic outlook of demyelination in aging, AD, and ischemic stroke in detail in the next sections.

## 3 DEMYELINATION IN AGING AND AD

Aging involves multifaceted WM integrity changes resulting in decreased cognitive functions. Magnetic resonance imaging examining myelin water fraction provided further evidence that changes in myelin serve as a sensitive indication of aging.^20^ Meanwhile, age‐related neurodegenerative diseases, such as AD, also present with overt demyelination,^6^, ^21^ lending credence to the use of myelin and OLs as potential therapeutic targets of aging and neurodegeneration.

### 3.1 Mechanisms of aging‐related myelin loss

**FIGURE 1:**
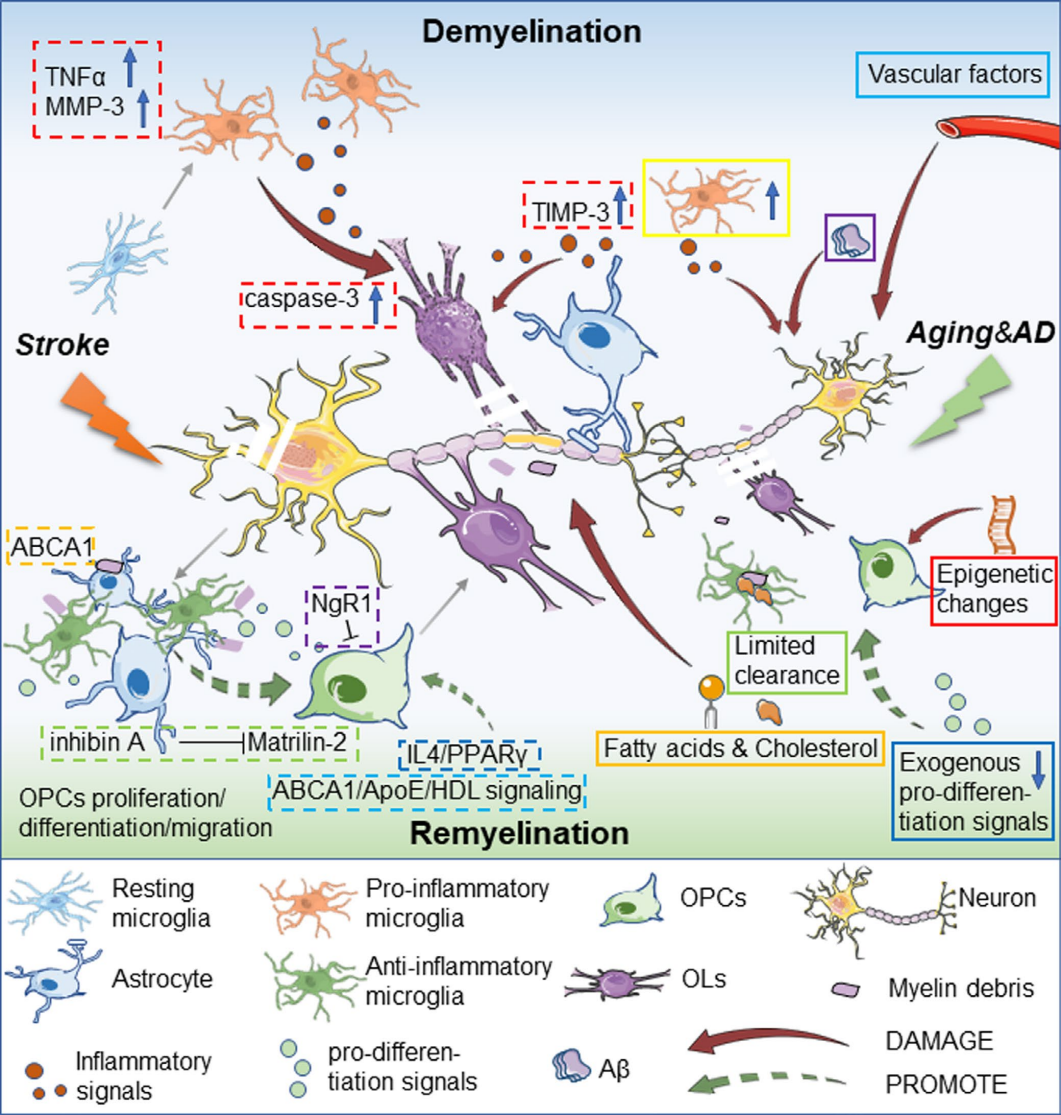
Demyelination and remyelination processes in aging, AD, and stroke. In aging and AD, vascular factors, microglial/astrocytic inflammatory factors, and β‐amyloid (Aβ) harms myelin integrity and OLs. Metabolism dysfunction involving fatty acids and cholesterol aggravate myelin breakdown. Epigenetic changes and absence of exogenous pro‐differentiation signals irreversibly burdens OPC proliferation/differentiation. Cholesterol limits microglia's clearance of myelin debris, which inhibits remyelination. In ischemic stroke, TIMP‐3 in astrocytes, and TNF‐α and MMP‐3 in microglia are induced, which increases caspase‐3 dependent OLs death and worsens demyelination injury. Microglia and infiltrating macrophages are activated to clear damaged myelin debris and dead neurons. Astrocytes also participate in the removal of dead cells and myelin debris through the ABCA1 pathway. Many molecular mechanisms are involved in remyelination after stroke. Reactive astrocytes secrete inhibin A and inhibit the expression of Matrilin‐2 in OPCs, which is not conducive to OPC differentiation and remyelination. NgR1 signaling is not conducive to the transformation of OPC into OLs. The cholesterol synthesis‐related ABCA1/ApoE/HDL signaling pathway promotes the migration of OPCs and the maturation of OLs after stroke. Interleukin‐4/PPARγ signal axis promotes OPC differentiation and maturation

Oligodendrocytes produce and support myelin, and therefore, loss and functional dysregulation of OLs result in myelin breakdown. OLs and OPCs are vulnerable to inflammation and DNA damage, as well as amyloid‐β (Aβ) accumulation that can lead to Aβ toxicity.^22^, ^23^ Thus, it is not surprising that degeneration of OLs and OPCs increase with aging,^24^ as these processes increase with aging. It is confirmed that epigenetic change causes a decline in the differentiation ability of OPCs with aging, which may cause dysfunction or absence of OLs^25^ (Figure 1 red square).

Loss of OLs is not the only underlying etiology for aging‐related demyelination. Indeed, myelin composition can be regulated in a manner that is independent of OL survival. Loss and degeneration of myelin proteins also contribute to demyelination in aging. Absence of the 21.5 kilodalton isoform of MBP, or age‐related dysregulation of 2′,3′‐cyclic‐nucleotide 3′‐phosphodiesterase (CNPase) may also disrupt myelin's structure.^26^ In addition to changes in proteins, alterations in cholesterol and fatty acids can cause myelin damage as observed in WM abnormality in AD,^21^ which is consistent with recent research that revealed lipid metabolism is essential for the maintenance of myelin integrity in adulthood^26^ (Figure 1 orange square). Notably, in AD, Aβ‐induced loss of MBP may contribute to the progression of the disease^27^ (Figure 1 purple square).

Other components of the aging brain's environment are also tightly linked to myelin homeostasis. As the resident immune cells in WM, the ability of microglia to clear myelin fragments is crucial for myelin homeostasis.^28^, ^29^ Microglia is thought to develop a pro‐inflammatory phenotype in the aged brain and to contribute to myelin loss^30^ (Figure 1 yellow square). A study has shown that aging‐related myelin degradation burdened the clearance function of microglia, which in turn contributed to microglial senescence and immune dysfunction in aging.^31^ Microglia and astrocytes both play a role in the formation and maintenance of myelin.^32^ Aging‐related changes in astrocytes have been studied for many years. Astrocytes facilitate myelination and myelin maintenance under healthy condition, while the increase of abnormal astrocytes in WM is related to the loss of myelin in AD and aging.^33^ Moreover, vascular‐related changes also affect WM aging^34^ (Figure 1 cyan square). Pericyte dysfunction leads to a variety of diseases associated with cognitive impairment, which has been found to be related to demyelination.^35^, ^36^

### 3.2 Deficiencies in remyelination with aging

As a counterbalance to demyelination, remyelination is a highly effective regenerative process and key regeneration mechanism for WM repair. Until recently, OL and myelin generation were thought to continue unencumbered throughout normal adulthood,^37^ as the great majority of myelinating OLs were presumed to retain stability throughout life.^37^ However, recent studies indicate that there is a decline in myelin plasticity with age, which may limit the efficiency of remyelination after injury. Indeed, remyelination occurs promptly after demyelination in a cuprizone‐induced demyelination mouse model using young animals, which included the re‐expression of myelin markers and the regeneration of OLs, but is compromised and incomplete in aged mice after treatment with cuprizone.^38^ Furthermore, demyelination related to neurodegeneration may stimulate oligodendrogenesis at early stages^39^ to facilitate remyelination, but this process may be hindered as the plasticity of OLs and myelin experience age‐related decline, resulting in worsening WM degeneration.

The impact of aging on remyelination is widespread. Aging has been reported to impair both OPC recruitment and differentiation, which may play a role in the age‐related decrease in remyelination.^40^ Hence, therapies targeting the regulation of OPCs and OLs regeneration and differentiation may be promising treatment strategies for demyelination.^41^ Björn Neumann et al recently explored metformin's potential to restore the regenerative capacity of aged OPCs, and observed reinstatement of remyelination as OPCs regained their sensitivity to pro‐differentiation signals in response to metformin.^42^ However, aging also restricts the ability of transplant mesenchymal stem cells to promote the generation of OLs during remyelination, which may limit stem cell therapies’ application in aging.^43^ This leads to a theory that the aging of environmental factors of OPCs may be to blame for deteriorating remyelination.

Microglia and astrocytes play a role in constructing the environment of OPCs. It is revealed that microglia and monocyte‐derived macrophages are critical for successful remyelination,^44^ and age‐associated changes in microglia and defective cholesterol clearance may play a vital role in limiting remyelination in the aged CNS^45^ (Figure 1 green square) given that aged OPCs maintain the potential to rejuvenate in response to exogenous pro‐differentiation signals^42^, ^46^ (Figure 1 blue square). Therefore, optimizing the microenvironment of OPCs via modulation of microglia and other factors is a potential remyelination‐support therapy. In support of this theory, Rawji et al recently found that niacin enhanced myelin phagocytosis by rejuvenating macrophage/microglia, which promoted OPCs recruitment and remyelination.^43^ The role that astrocytes play in the aging‐related decline in remyelination remains to be clearly elucidated.

In the end, age‐related deficiencies in remyelination may be unavoidable as it may be regulated by age‐dependent epigenetic control.^47^ However, as our knowledge of the inhibitory mechanism(s) underlying the negative impact of aging on remyelination deepens, so will the prospects of remyelination therapies to counter these effects. Targeting both OPCs’ vitality and their aging microenvironment holds promise for future therapies to combat aging‐related demyelination.

### 3.3 Myelin‐related changes in Alzheimer's disease

During the early stages of AD, the loss of WM is one of the pathological changes observed, often preceding the presence of the hallmark neurofibrillary tangles and plaques that underlie neuronal deficits.^48^ The molecular mechanism of myelin loss in AD has not been fully elucidated, but it may include oxidative stress, neuroinflammation, and excitotoxicity.^48^ It has been suggested that a vicious cycle of myelin loss and failure of regeneration from OPCs plays an important role in AD.^48^ This cycle purportedly involves neurotoxic Aβ accumulation leading to myelin damage^27^; however, paradoxically it has been recently reported that Aβ can also promote oligodendrocyte differentiation and maturation.^49^ Although these two actions of Aβ are contradictory, it is undeniable that the early changes in oligodendrocytes and myelin precede the deposition of Aβ.^39^, ^50^

Consistent with aging‐related demyelination, AD‐associated demyelination involves both OPCs dysfunction and environmental remyelination inhibition. Defective microglial phagocytosis also plays a role in AD pathology.^51^ This leads us to posit that the gradual degeneration or aging of the brain microenvironment may be responsible for the irreversible demyelination by disrupting OPCs’ renewal processes and myelin integrity. Therefore, concentrated focus on mechanisms responsible for early OPCs and myelin dysfunction, which may lead to therapies that mitigate the hostile brain microenvironment and microglia phagocytosis dysfunction, hold promise to treat both age‐related neurodegeneration and AD by promoting remyelination.

## 4 DEMYELINATION AND REMYELINATION IN STROKE

Stroke is a major cause of death and disability worldwide and it causes increasing health and economic burdens globally, especially in low‐ and middle‐income countries.^52^ Stroke risk varies by age, gender, race, and geographical location. It is widely accepted that many risk factors are associated with stroke, including aging, smoking, drinking, hypertension, diabetes, hyperlipidemia, sleep patterns, and so on.^53^ Age is one of the most important risk factor for stroke as the risk of stroke increases dramatically with age in both males and females.^54^ In addition, remyelination in WM lesions becomes less efficient with age.^38^, ^40^ As the world's population continues to age, the prevalence of stroke will further increase.^55^ Ischemic stroke is often accompanied by WM injury. WM constitutes half of the human brain and is more vulnerable to ischemic injury than gray matter, so WM damage is a key component of ischemic damage.^56^, ^57^, ^58^ WM damage caused by ischemic stroke leads to long‐term sensorimotor and cognitive impairment. Although there have been a large number of preclinical and clinical trials on stroke, there have been few studies on WM damage and recovery after stroke.

### 4.1 Myelin sheath loss after stroke

WM is mainly composed of myelinated and unmyelinated axons, OLs, and other glial cells (including microglia and astrocytes) in the CNS. In order to maintain the normal function of neurons, the myelin sheaths need to persist throughout adulthood. Mature myelin lipids are rapidly metabolized. To maintain the integrity of myelin in adulthood, continuous synthesis and metabolism of myelin components are required.^59^ OLs synthesize myelin in the CNS, which are extremely susceptible to ischemic stroke.^57^ Ischemic stroke is accompanied by the destruction of myelin structure and apoptosis of OLs.^7^, ^60^ Demyelination is thought to be the result of OL death caused by endogenous or exogenous injury. Recent studies have shown that in addition to the death of OLs, decreased levels of myelin lipids may also be the cause of demyelination. Myelin lipid metabolism disorders are sufficient to drive demyelination independent of the death of OLs.^59^ Cholesterol synthesis and transport disorders reduce myelination, increase OL loss, and decrease oligodendrogenesis in the ischemic brain after stroke.^61^

On the other hand, ischemic stroke induces the proliferation of OPCs, which can proliferate and differentiate into mature OLs in an attempt to restore OLs.^62^ As mentioned previously, remyelination is one of the key regeneration mechanisms of WM repair. However, most of these OPCs are stopped in the early stages of differentiation into mature OLs, therefore failed to differentiate into mature OLs, and even a small amount of the proliferating OPCs differentiate into astrocytes, resulting in incomplete remyelination.^63^, ^64^ Although it is still controversial whether astrocytes and microglia play a beneficial or detrimental role in the process of remyelination, it appears they are essential to promoting the proliferation and differentiation of OLs and remyelination of axons.^65^ Subcortical WMS accounts for 25% of all stroke subtypes, and its pathological hallmark is loss of myelin sheaths.^8^ WMS manifest as OL death, myelin sheath loss, axonal degeneration, microglia activation, WM atrophy, and functional impairment, and age will further aggravate these injuries.^66^

### 4.2 Neuroinflammation affects the demyelination and remyelination of OLs

Inflammation occurs within minutes or hours after ischemia stroke and impacts the severity of damage and neurological deficits in stroke patients.^67^ Limiting the early inflammatory response after ischemic stroke may be a feasible way to improve stroke outcome. Microglia and astrocytes are the innate immune cells in the brain, which are rapidly activated after ischemic stroke.^68^ After ischemic stroke, microglia quickly activate in response to dead neurons, subsequently releasing inflammatory factors, and causing an inflammatory response.^69^, ^70^ Activated microglia are considered to be the main source of inflammatory factors.^71^ It is worth noting that the pro‐inflammatory/anti‐inflammatory phenotypes of microglia in the brain after stroke are not static. The two phenotypes of microglia will transform into each other as the change of microenvironmental signals in the brain. Studies on mouse stroke models revealed that in the initial stage of ischemic stroke, the microglia in the peripheral area of the injury mainly showed an anti‐inflammatory phenotype, and gradually transformed into pro‐inflammatory microglia dominating the landscape weeks after injury.^72^, ^73^ In addition to microglia, astrocytes are also an important part of the brain's innate immune system, which are activated and proliferate after stroke.^69^ Both microglia and astrocytes exhibit dual roles in ischemic injury. Pro‐inflammatory microglia release pro‐inflammatory factors, which aggravate the inflammatory response caused by ischemia and thus aggravate ischemic damage. In contrast, anti‐inflammatory microglia phagocytose dead cell debris and release neuroprotective factors that play a protective role in ischemic injury.^74^ The anti‐inflammatory microglia is known to promote oligodendrogenesis and remyelination. Similarly, A1‐reactive astrocytes produce and release pro‐inflammatory mediators to aggravate the death of neurons and OLs. In contrast, A2‐reactive astrocytes are assumed to have a neuroprotective effect by releasing neuroprotective factors in the peripheral area of ischemia to support neuron survival.^69^

Studies have shown that increasing the number of anti‐inflammatory microglia in an ischemic stroke model can promote OPC differentiation and remyelination.^75^, ^76^ On the contrary, the inflammatory response of astrocytes and microglia induced in ischemic brain leads to the death of immature OL. Studies suggest that ischemic stroke induces high expression of tissue inhibitors of metalloproteinase‐3 (TIMP‐3) in astrocytes and promotes high expression of tumor necrosis factor‐α (TNF‐α) and matrix metalloproteinase‐3 (MMP‐3) in microglia, promoting inflammation in the ischemic brain, thereby increasing the death of caspase‐3 dependent immature OLs^7^ (Figure 1 red dotted square).

It is now well known that anti‐inflammatory microglia and infiltrated macrophages clear damaged myelin debris in ischemic stroke, which possibly facilitates remyelination.^77^ Although it is traditionally believed that pro‐inflammatory microglia impairs the regeneration of OLs in a TNF‐α‐dependent manner, studies have also revealed that pro‐inflammatory phagocytosis of microglia, especially the expression of TNF‐α after demyelination injury, is necessary to initiate the regenerative response for myelin repair, including the clearance of myelin debris and the regeneration of OLs.^78^, ^79^ This is consistent with the fact that TNF‐α is considered to be a pro‐inflammatory factor with a dual role.^80^ Astrocytes are also involved in phagocytosis and the removal of dead cells and myelin structural fragments after ischemic stroke.^8^, ^81^ ATP‐binding cassette transporter A1 (ABCA1) pathway is one of the main molecular mechanism for astrocytic phagocytosis^8^, ^81^ (Figure 1 orange dotted square).

### 4.3 Remyelination and its molecular signaling pathway in stroke

Oligodendrocyte precursor cells accumulate and proliferate in the peri‐infarct area in response to stroke. In a study of WM stroke, it was found that <10% of those OPCs differentiate into mature OLs and 4%‐13% of those OPCs differentiate into astrocytes, while the remaining proliferating OPCs are locked in the progenitor cell phenotype.^63^ Therefore, insufficient proliferation, migration, and differentiation of OPCs may be the cause of insufficient remyelination in stroke. Although the molecular mechanisms that inhibit OPC differentiation are not well understood, several molecular pathways may be involved. Nogo is a signal molecule that was discovered relatively early and plays an important role in regulating the myelination of OLs during development.^82^ Studies have shown that blocking Nogo receptor 1 (NgR1) signaling promote the conversion of OPC into OLs in a WMS model, suggesting that NgR1 signaling partly participates in the obstruction of OPC differentiation after stroke^63^ (Figure 1 purple dotted square).

In addition, astrocytes are also involved in the regulation of OPC differentiation after stroke. Studies have also shown that after stroke, reactive astrocytes secrete inhibin A and inhibit the expression of Matrilin‐2 in OPC, which creates a microenvironment that is not conducive to OPC differentiation and remyelination^83^ (Figure 1 green dotted square). Moreover, cholesterol is the lipid component that makes up myelin. Blocking cholesterol synthesis‐related pathways, such as the ATP‐binding cassette transporter A1 (ABCA1)/apolipoprotein E (ApoE)/high‐density lipoprotein (HDL) signaling pathway, reduces the migration of OPC, the maturation of OLs, and myelination in the ischemic brain after stroke^61^ (Figure 1 cyan dotted square).

In recent years, some treatments in animal models have proven to promote remyelination after demyelination. Omega‐3 polyunsaturated fatty acids (n‐3 PUFAs) likely contribute to OL survival and promote oligodendrogenesis in a mouse stroke model^84^ and notably decrease demyelination and promote functional recovery after stroke in aged mice.^85^ Interleukin‐4/peroxisome proliferator‐activated receptor gamma (PPARγ) signal axis promotes OPC differentiation and maturation and improves WM integrity after stroke^75^ (Figure 1 blue dotted square). Similarly, the PPARγ agonist rosiglitazone was found to promote oligodendrogenesis after ischemic stroke.^86^ These studies suggest that PPARγ is a potential target to promote the differentiation and maturation of OPC after stroke. Inhibition of CD147 which is known as a key mediator that inhibits inflammation and immune response also can increase the proliferation and maturation of OPC after stroke.^87^ Recent studies indicate that motor learning promoted oligodendrogenesis, OPC differentiation, and new myelin sheaths generation in cuprizone‐induced demyelination.^88^ On the other hand, motor learning did not change the survival of mature OLs but promoted the remyelination of surviving mature OLs. This study described a novel pattern of remyelination: most of the newly formed myelin sheaths wrap previously unmyelinated axons (remodeling) rather than the demyelinated axons (remyelinating).^88^

## 5 THERAPEUTIC PROSPECT FOR DEMYELINATING DISEASES

Demyelination connects many CNS diseases as a common pathological characteristic resulting in neurological dysfunction, which desires more our concentration. Delightingly, the immunotherapies of MS, the most typical demyelinating disease, have been transformed successful.^89^ However, it is still challenging to prevent the progression of MS via remyelination therapy though more and more clinical trials have transferred their focus on myelin repair. Despite the great number of remyelination‐support drugs, little have been applied in phase 2 clinical trials so far.^89^ Moreover, our great attention of remyelination therapies was restricted in MS all along before. Although the major cause of demyelination varies in MS, aging, ischemia stroke, and other demyelinating diseases, understanding the heterogeneity of demyelination and remyelinating drawback is hopefully helpful for inventing advanced and universal remyelination therapies. Notably, a very large number of drugs which enjoy successful pro‐myelinating potential in OPCs culture have failed in animal models or clinical trials, suggesting there lies huge obstacles resisting simple remyelination therapies. Hopefully, newly emerging studies arise focusing more on environment‐targeting or multi‐targeting therapeutic strategies.

Oligodendrocytes and OPCs display an impressive capacity for regeneration, differentiation, and myelin repair^90^ in rudimentary models of demyelination. Therefore, promoting OLs and OPCs proliferation and differentiation are obvious targets for therapeutic intervention to facilitate remyelination in a myriad of demyelination‐related diseases. However, in more complex models that are more clinically relevant, numerous impediments to OL remyelination exist, including epigenetics factors and environmental deterioration, which threaten to derail the application of OL proliferation promoting therapies. Based on these insights, emerging pro‐remyelination therapeutic strategies targeting the CNS environment have been examined that show promise (Table 1). Remyelination therapy offers the possibility of improving both demyelinating diseases and aging and thus warrants further investigation.

**TABLE 1:** Emerging therapeutic prospect of remyelination in aging and demyelinating diseases

Therapy	Target	Model	Reference
Mesenchymal stem cells transplantation	Oligodendrocytes proliferation	Mouse EAE model and MS model	87
Stem cell‐derived extracellular vesicles	Oligodendrocytes proliferation	Rat subcortical stroke model and in vitro OGD model	88
Fasting or metformin	Oligodendrocytes proliferation	Aged rat focal demyelination model	42
NF155 overexpression	Oligodendrocytes differentiation and myelin repair	Rat in vitro hypoxic‐ischemic mixed cell model	89
miR‐125a‐3p silencing	Oligodendrocytes differentiation and myelin repair	Mouse in vivo and ex vivo lysolecithin‐induced demyelination model	90
miR‐17‐92 enriched exosomes	Oligodendrocytes differentiation and myelin repair	Rat tMCAO ischemia stroke model	91
n‐3 PUFAs supplementation	Oligodendrocytes differentiation and myelin repair	Mouse ischemic stroke (MCAO) model	81, 82
Interleukin‐4 treatment	Oligodendrocytes differentiation and myelin repair	Mouse ischemic stroke (MCAO) model	59
Rosiglitazone	Oligodendrocytes differentiation and myelin repair	Mouse ischemic stroke (MCAO) model	83
Inhibition of CD147	Oligodendrocytes differentiation and myelin repair	Mouse ischemic stroke (MCAO) model	84
CX3CR1 antibody	Microglia inhibition	Mouse brain ischemic model	92
Niacin	Microglia rejuvenation	Aged mouse and microglia cultures	77
Minocycline plus N‐acteylcysteine	Microglia/macrophage polarization regulation	Rat mild mCCI model of traumatic brain injury	93
Growth differentiation factor‐11 supplementation	Angiogenesis	Aged mouse ischemic stroke (MCAO) model	94
Environmental enrichment	Others	Mouse perinatal hypoxia model	95
Youthful blood exchange	Others	Mouse focal demyelinating spinal cord lesion model	46
Motor learning	Others	cuprizone‐induced demyelination model	85
Mesenchymal stem cells transplantation	Oligodendrocytes proliferation	Mouse EAE model and MS model	91
Stem cell‐derived extracellular vesicles	Oligodendrocytes proliferation	Rat subcortical stroke model and in vitro OGD model	92
Fasting or metformin	Oligodendrocytes proliferation	Aged rat focal demyelination model	42
NF155 overexpression	Oligodendrocytes differentiation and myelin repair	Rat in vitro hypoxic‐ischemic mixed cell model	93
miR‐125a‐3p silencing	Oligodendrocytes differentiation and myelin repair	Mouse in vivo and ex vivo lysolecithin‐induced demyelination model	94
miR‐17‐92 enriched exosomes	Oligodendrocytes differentiation and myelin repair	Rat tMCAO ischemia stroke model	95
n‐3 PUFAs supplementation	Oligodendrocytes differentiation and myelin repair	Mouse ischemic stroke (MCAO) model	84, 85
Interleukin‐4 treatment	Oligodendrocytes differentiation and myelin repair	Mouse ischemic stroke (MCAO) model	75
Rosiglitazone	Oligodendrocytes differentiation and myelin repair	Mouse ischemic stroke (MCAO) model	86
Inhibition of CD147	Oligodendrocytes differentiation and myelin repair	Mouse ischemic stroke (MCAO) model	87
CX3CR1 antibody	Microglia inhibition	Mouse brain ischemic model	96
Niacin	Microglia rejuvenation	Aged mouse and microglia cultures	77
Minocycline plus N‐acetylcysteine	Microglia/macrophage polarization regulation	Rat mild mCCI model of traumatic brain injury	97
Growth differentiation factor‐11 supplementation	Angiogenesis	Aged mouse ischemic stroke (MCAO) model	98
Environmental enrichment	Others	Mouse perinatal hypoxia model	99
Youthful blood exchange	Others	Mouse focal demyelinating spinal cord lesion model	46
Motor learning	Others	cuprizone‐induced demyelination model	88

## CONFLICT OF INTEREST

The authors declare no conflict of interest.

## AUTHOR CONTRIBUTIONS

YGao designed the review. DC, YH, ZS, JL, YZ, KW, and YGong wrote the manuscript. DC and YH drew the figures. AS, YGong, and YGao critically edited the manuscript.

## Data Availability

This review manuscript has no original data. The authors confirm the absence of shared data.
